# Occurrence and distribution of sarcoptic mange in wild Neotropical canids

**DOI:** 10.1111/mve.70085

**Published:** 2026-05-04

**Authors:** Luan de Jesus Matos de Brito, Guilherme S. T. Garbino

**Affiliations:** ^1^ Laboratório de Mastozoologia, Museu de Zoologia João Moojen, Departamento de Biologia Animal Universidade Federal de Viçosa Viçosa Minas Gerais Brazil; ^2^ Programa de Pós‐graduação em Biologia Animal Universidade Federal de Viçosa Viçosa Minas Gerais Brazil

**Keywords:** host–parasite interactions, *Sarcoptes scabiei*, skin disease, systematic review, vector‐borne pathogens, wildlife disease, zoonosis

## Abstract

Sarcoptic mange, a contagious skin disease caused by *Sarcoptes scabiei*, is among the most widespread parasitic infections in mammals. Despite its global importance, its effects on wild Neotropical canids have received limited scientific attention. We aimed to compile and analyse all available records of sarcoptic mange in Neotropical canids to assess its geographical distribution, host range and potential conservation implications. We conducted a comprehensive literature review of published and unpublished reports describing cases of sarcoptic mange in wild Neotropical canids across their distribution range. Our synthesis revealed that sarcoptic mange affects more than two‐thirds of Neotropical canid species and occurs in at least six countries. Most records originated from areas dominated by dry and open forest formations, suggesting potential environmental associations with disease occurrence. These findings indicate that sarcoptic mange represents a neglected but widespread health issue in Neotropical carnivores. The disease's broad distribution and cross‐species occurrence raise concerns about population‐level impacts and the potential for interspecific transmission. We propose recognizing sarcoptic mange as an emerging threat to Neotropical canid conservation. To mitigate its effects, we recommend implementing systematic monitoring programmes, including screening of apparently healthy individuals and genetic characterization of mites to trace transmission pathways. Understanding this host–parasite interface is crucial for predicting and managing the broader ecological and epidemiological consequences of mange in the region.

## Graphical abstract

- Sarcoptic mange affects most Neotropical canid species across multiple countries.
- It represents a widespread yet largely overlooked conservation threat with potential for cross‐species transmission.
- Coordinated monitoring and management efforts are needed to understand and mitigate its impacts.

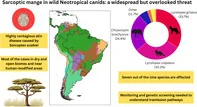

## INTRODUCTION

Emerging infectious diseases in wildlife represent a major global threat, often driving severe population declines and even local extirpations (Daszak et al., 2000; Soulsbury et al., 2007; Tompkins et al., 2015). Among these, sarcoptic mange, caused by the parasitic mite *Sarcoptes scabiei* (Linnaeus, 1758), has been increasingly recognized as an important disease affecting wildlife populations worldwide. It can cause severe morbidity and mortality in a wide range of mammalian hosts and also poses risks to humans (Escobar, 2022; Karimkhani et al., 2017; Montecino‐Latorre et al., 2020).

In canids, infection may result in severe clinical manifestations such as extensive skin lesions, alopecia, emaciation, overall poor body condition and, in advanced cases, mortality (Fiori et al., 2025; Newman et al., 2002; Verdugo et al., 2016). The disease has been linked to population declines in several carnivore species, with cascading effects on ecosystem dynamics and biodiversity conservation (Guillot et al., 2023; Niedringhaus et al., 2019; Soulsbury et al., 2007). In the Neotropics, where canid diversity is remarkable, sarcoptic mange poses a potential threat to population health, particularly in species already exposed to anthropogenic and environmental pressures (Acosta‐Jamett & Napolitano, 2024; Montecino‐Latorre et al., 2020).

Despite its recognized importance in wildlife health, the epidemiology of sarcoptic mange in Neotropical canids remains poorly understood (Anello et al., 2025). Most available records consist of case reports or short communications, which, although valuable, do not provide a comprehensive picture of spatial, temporal or taxonomic patterns. The lack of a systematic synthesis hampers the identification of hotspots, the detection of temporal trends and the assessment of species‐specific vulnerability, all of which are crucial for effective conservation and management strategies (Gortázar et al., 2025).

Here, we conducted a systematic literature review to compile and analyse reports of *S. scabiei* infection in wild Neotropical canids. Specifically, we aimed to (i) characterize the spatial and temporal distribution of cases, (ii) identify the most affected species and diagnostic approaches employed and (iii) synthesize the ecological impacts reported. By consolidating scattered information, this study provides an updated overview of sarcoptic mange in Neotropical canids, offering a baseline for future research and conservation planning.

## MATERIALS AND METHODS

We conducted a systematic literature review between August and September 2025, following PRISMA guidelines (Page et al., 2021), to identify and compile case reports and short communications documenting *S. scabiei* infection in wild Neotropical canids. Searches were performed in SciELO, Scopus and Web of Science (WoS) databases, targeting titles, abstracts and keywords. Boolean operators were used to combine terms related to the disease, host group and ecological context. The complete search string used across databases was: (“*Sarcoptes scabiei*” OR “sarcoptic mange” OR scabies OR sarna OR escabiose) AND (“Canidae” OR “wild canid” OR “wild dogs” OR canídeo OR “Cerdocyon thous” OR “Chrysocyon brachyurus” OR “Lycalopex culpaeus” OR “Lycalopex griseus” OR “Lycalopex gymnocercus” OR “Lycalopex sechurae” OR “Lycalopex vetulus” OR “Atelocynus microtis” OR “Speothos venaticus” OR “graxaim” OR “lobo‐guará” OR “raposa” OR “cachorro‐do‐mato” OR “cachorro‐vinagre” OR “crab‐eating fox” OR “maned wolf” OR “culpeo fox” OR “Pampas fox” OR “South American gray fox” OR “Sechura fox” OR “hoary fox” OR “short‐eared dog” OR “bush dog” OR “zorro” OR “perro salvaje” OR “renard” OR “chien sauvage” OR “vos” OR “wilde hond”) AND (“mammal*” OR “animal*” OR “wildlife” OR “fauna”)**.

Additionally, common and scientific names of all extant Neotropical canid species were included in Portuguese, Spanish, English, French and Dutch to ensure comprehensive taxonomic coverage and mitigate geographical bias. Any discrepancies during the screening and eligibility phases were resolved through collective consensus among the authors, ensuring that all documented reports retrieved by the filters were rigorously evaluated for inclusion.

We included studies published between 2002 and October 2025, in Portuguese, Spanish, English, French or Dutch, provided they reported natural infections of *S. scabiei* in wild Neotropical canids. Exclusion criteria comprised studies involving domestic animals, laboratory experiments or non‐native populations. For each eligible record, we extracted host species, year of publication, geographic location, diagnostic method and ecological impacts described. Besides the systematic literature review, we also manually searched the databases and available compendia.

When geographical coordinates were provided in the publication, we used them directly; when only the county was reported, we used approximate coordinates. Based on these locations, we extracted the predominant biome using the classification of Olson et al. (2001). We also categorized the surrounding environment as one of the following: rural area (plantations or cattle ranches), periurban area (near urban centres) or near villages (close to small settlements). All analyses and figures were produced in the R environmental (R Core Team, 2023) using the ggplot2 package (Wickham, 2016).

During the full‐text screening of the articles retrieved in the systematic review, we identified four additional relevant studies through citation tracking. These studies were not retrieved by the systematic search, likely because they fall outside the scope of the indexed sources queried. Given that they provide clear evidence of scabies in Neotropical canids, they were included in the review.

## RESULTS

Following the PRISMA guidelines, we initially identified 88 studies (Garbino, 2026). After removing 17 duplicates, 71 records remained for title screening, of which 43 were excluded based on their titles (Figure S1). The remaining 28 studies were evaluated in full text. Among these, eight did not involve natural infection, six involved only domestic animals and six reported secondary accounts of previously published data and were therefore excluded. Ultimately, eight studies met the inclusion criteria and were included in this review (Table 1).

**TABLE 1:** Localities, arranged latitudinally, of the sarcoptic mange cases in Neotropical canids found in this review, as well as the host species, common name and diagnostic method.

#ID	Locality	State/province	Country	Coordinates	Precision	Biome	Environment	Host	Diagnostic method	References
1	Duran	Guayas	Ecuador	02°13′17″S, 79°42′06″W	Precise	Tropical dry broadleaf forest	Periurban area	Sechuran Fox (*Lycalopex sechurae*)	Clinical condition and visual identification	Villalba‐Briones et al. (2022)
2	Huaral	Huaral	Peru	11°25′38″S, 77°10′46″W	Precise	Xeric shrubland	Periurban area	Culpeo (*Lycalopex culpaeus*)	Visual identification and molecular identification	Gomez‐Puerta et al. (2024)
3	Ventanilla	Callao	Peru	11°49′06″S, 77°06′24″W	Precise	Xeric shrubland	Urban area	Culpeo (*Lycalopex culpaeus*)	Visual identification and molecular identification	Gomez‐Puerta et al. (2024)
4	Victoria Ranch	Beni	Bolivia	13°37′S, 65°07′W	Precise	Tropical savanna	Rural area	Maned Wolf (*Chrysocyon brachyurus*)	Clinical condition	Díaz Luque et al. (2014)
5	Nova Xavantina	Mato Grosso	Brazil	14°40′S, 52°21′W	Estimated	Tropical savanna	Rural area	Bush Dog (*Speothos venaticus*)	Clinical condition and visual identification	Jorge et al. (2008), Jorge and Jorge (2014)
6	Charagua	Santa Cruz	Bolivia	19°10′43″S, 62°31′33″W	Precise	Tropical dry broadleaf forest	Near village	Pampas Fox (*Lycalopex gymnocercus*)	Clinical condition and visual identification	Deem et al. (2002)
7	Belo Oriente	Minas Gerais	Brazil	19°13′S, 42°29′W	Estimated	Tropical moist broadleaf forest	Rural area	Crab‐eating Fox (*Cerdocyon thous*)	Clinical condition and visual identification	de Oliveira et al. (2023)
8	Charagua	Santa Cruz	Bolivia	19°38′07″S, 62°39′24″W	Precise	Tropical dry broadleaf forest	Near village	Pampas Fox (*Lycalopex gymnocercus*)	Clinical condition and visual identification	Deem et al. (2002)
9	Lavras	Minas Gerais	Brazil	21°14′S, 45°00′W	Estimated	Tropical moist broadleaf forest	Rural area	Crab‐eating Fox (*Cerdocyon thous*)	Clinical condition and visual identification	Teodoro et al. (2018)
10	Cajuru	São Paulo	Brazil	21°16′S, 47°18′W	Estimated	Tropical savanna	Not specified	Maned Wolf (*Chrysocyon brachyurus*)	Clinical condition and visual identification	Fiori et al. (2023)
11	Mococa	São Paulo	Brazil	21°28′S, 47°00′W	Estimated	Tropical savanna	Not specified	Maned Wolf (*Chrysocyon brachyurus*)	Clinical condition and visual identification	Fiori et al. (2023)
12	Santo Antônio de Pádua	Rio de Janeiro	Brazil	21°32′S, 42°10′W	Estimated	Tropical moist broadleaf forest	Not specified	Maned Wolf (*Chrysocyon brachyurus*)	Clinical condition and visual identification	Fiori et al. (2025)
13	São José do Rio Pardo	São Paulo	Brazil	21°35′S, 46°53′W	Estimated	Tropical moist broadleaf forest	Not specified	Maned Wolf (*Chrysocyon brachyurus*)	Clinical condition and visual identification	Fiori et al. (2023)
14	São João da Boa Vista	São Paulo	Brazil	21°58′S, 46°47′W	Estimated	Tropical savanna	Not specified	Maned Wolf (*Chrysocyon brachyurus*)	Clinical condition and visual identification	Fiori et al. (2023)
15	São Carlos	São Paulo	Brazil	22°00′S, 47°53′W	Estimated	Tropical savanna	Not specified	Maned Wolf (*Chrysocyon brachyurus*)	Clinical condition and visual identification	Fiori et al. (2025)
16	Itirapina	São Paulo	Brazil	22°15′S, 47°49′W	Estimated	Tropical savanna	Not specified	Maned Wolf (*Chrysocyon brachyurus*)	Clinical condition and visual identification	Fiori et al. (2025)
17	Mogi Guaçu	São Paulo	Brazil	22°22′S, 46°56′W	Estimated	Tropical savanna	Not specified	Maned Wolf (*Chrysocyon brachyurus*)	Clinical condition and visual identification	Fiori et al. (2023)
18	Itatiba	São Paulo	Brazil	23°00′S, 46°50′W	Estimated	Tropical moist broadleaf forest	Not specified	Maned Wolf (*Chrysocyon brachyurus*)	Clinical condition and visual identification	Jorge and Jorge (2014), Fiori et al. (2023)
19	Atibaia	São Paulo	Brazil	23°07′S, 46°33′W	Estimated	Tropical moist broadleaf forest	Periurban area	Crab‐eating Fox (*Cerdocyon thous*)	Clinical condition and visual identification	Jorge and Jorge (2014)
20	Undetermined	Ñuble	Chile	36°36′S, 72°07′W	Estimated	Mediterranean scrub	Rural area	Culpeo (*Lycalopex culpaeus*) and South American Grey Fox (*L. griseus*)	Clinical condition and visual identification	Millán et al. (2024)
21	Osorno	Los Lagos	Chile	40°34′S, 73°07′W	Estimated	Temperate coniferous forest	Rural area	South American Grey Fox (*L. griseus*)	Visual identification and molecular identification	Verdugo et al. (2016)
22	Campina do Monte Alegre	São Paulo	Brazil	23°33′35″S, 48°29′52″W	Precise	Tropical savanna	Rural area	Crab‐eating Fox (*Cerdocyon thous*)	Clinical condition	Oliveira et al. (2025)
23	Colina	Chacabuco	Chile	33°11′24″S, 70°43′12″W	Estimated	Mediterranean scrub	Rural area	Culpeo (*Lycalopex culpaeus*) and South American Grey Fox (*L. griseus*)	Clinical condition and visual identification	Millán et al. (2024)
Not mapped	Not specified	Mendoza	Argentina	Not mapped	Not mapped	Xeric shrubland	Not specified	Crab‐eating Fox (*Cerdocyon thous*)	Visual identification	Fain (1968)
Not mapped	Not specified	Paraíba	Brazil	Not mapped	Not mapped	Unknown	Not specified	Crab‐eating Fox (*Cerdocyon thous*)	Clinical condition and visual identification	Almeida et al. (2018)

*Note*: The biome classification follows Olson et al. (2001). Locality #ID refers to the localities in (Figure 1a). “Visual identification” refers to confirmation by skin scraping and direct observation of *Sarcoptes scabiei* under a stereomicroscope.

In addition, we incorporated four relevant studies identified outside the systematic search. These records were not retrieved by the database search because they fall outside the scope of the indexed sources queried. One of them is a chapter published in a Portuguese veterinary medicine textbook (Jorge & Jorge, 2014), and the other three were published in Brazilian journals that are not indexed in the databases searched (Almeida et al., 2018; Díaz Luque et al., 2014; Oliveira et al., 2025). Such records were identified through complementary screening of the literature and added to ensure completeness. In total, we examined 12 publications (Table 1).

In total, 85 case reports of sarcoptic mange were identified across twelve publications and five South American countries: Bolivia, Brazil, Chile, Ecuador and Peru (Figure 1a,b). Most of the records originated from Chile (58%), followed by Brazil (35%), with Bolivia, Ecuador and Peru representing less than 4% of the cases each. The disease was detected in seven out of the nine species (77%) of Neotropical canids (Figure 1d). None of the recorded species are globally classified as threatened by the IUCN, but *Chrysocyon brachyurus* (Illiger, 1815) and *Speothos venaticus* (Lund, 1842) are both classified as Vulnerable in the Brazilian Red List (Ministério do Meio Ambiente, 2022). We also found an early report of sarcoptic mange in *Cerdocyon thous* (Linnaeus, 1766) from Mendoza, Argentina, but excluded it from our analysis, as we considered it an isolated observation made approximately 40 years prior to the next recorded case (Fain, 1968).

**FIGURE 1:**
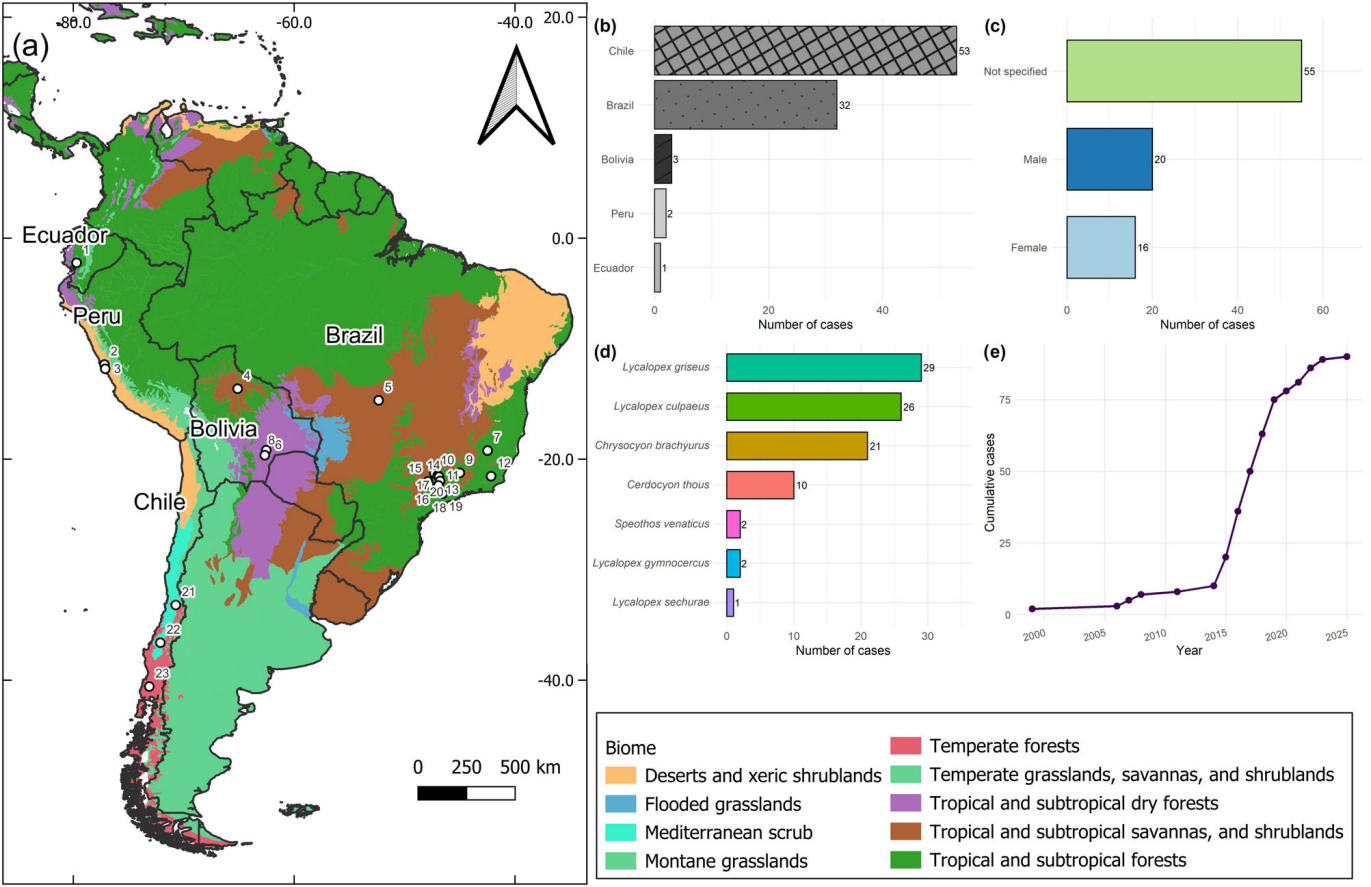
(a) Spatial distribution of sarcoptic mange recorded in wild Neotropical canids (for locality information see Table 1). Biome classification follows Olson et al. (2001); (b) Distribution of cases per country, (c) according to sex and (d) to species; (e) cumulative cases of sarcoptic mange in wild Neotropical canids between 1999 and 2025. Cases were pooled for the species: *Chrysocyon brachyurus*, *Cerdocyon thous*, *Lycalopex culpaeus*, *L. griseus*, *L. gymnocercus* and *L. sechurae*.

In most of the studies (92.86%), detection was made by skin scraping and visually identifying the mites, and also by molecular detection, but 7.14% of the studies diagnosed sarcoptic mange based only on the clinical condition of the individuals. Considering the individuals mentioned in the publications, a total of 29 animals underwent clinical treatment: 20 *Chrysocyon brachyurus*, seven *Cerdocyon thous*, one *Lycalopex sechurae* (Thomas, 1900) and one *Speothos venaticus*. All showed signs of recovery except for one *C. thous*, from Atibaia (Brazil), which died shortly after treatment began (Jorge & Jorge, 2014). One *Lycalopex culpaeus* (Molina, 1782) was found in critical condition and was euthanized, and two *C. thous*, one *L. griseus* (Grey, 1837) and one *S. venaticus* respectively died soon after clinical examination. Six individuals were found dead, including one *L. culpaeus*, two *L. gymnocercus* (Fischer, 1814) and one *C. thous*.

Treatment protocols varied among the studies. The female *S. venaticus* received two applications of topical selamectin (6 mg/kg) administered 35 days apart (Jorge & Jorge, 2014). Two *C. brachyurus* individuals were treated with Bravecto® (25–65 mg/kg) (Fiori et al., 2023). One *C. thous* received doramectin (0.6 mg/kg), followed seven days later by one tablet of afoxolaner 28.3 mg (NexGard® 4.1–10 kg) (Oliveira et al., 2023). In addition, adult individuals of *C. brachyurus* and *C. thous* pups in Brazil and adult *Lycalopex sechurae* in Chile were treated with ivermectin (0.4 mg/kg for *C. brachyurus* and *L. sechurae* and 200 μg/kg for the *C. thous* pups) (Almeida et al., 2018; Jorge & Jorge, 2014; Villalba‐Briones et al., 2022). In all reported cases, treated individuals showed signs of recovery.

Interestingly, most of the recorded cases come from relatively dry and open formations, such as the Beni savanna, Brazilian Cerrado, Chaco dry forests and Chilean Matorrales (Figure 1a, Table 1). Exceptions include the records from the Los Lagos region in Chile, a record from Rio de Janeiro state in Brazil and some records from savanna/forest transitional areas in São Paulo state (Table 1). Sex was not reported for the majority of cases (Figure 1c), and the low number of specimens with identified sex precludes making assumptions on sex‐biased contagion.

Temporal distribution of mange cases revealed marked variation across species and years, showing a sharp increase in number of cases after 2014 (Figure 1d). The earliest report came from 1968 in *Cerdocyon thous* (Fain, 1968), and only in 1999 were new cases reported for *L. gymnocercus* (Deem et al., 2002). In 2006 and 2008 the first cases were published for *Chrysocyon brachyurus*, which maintained a low but continuous frequency until 2023. In *Cerdocyon thous*, cases reappeared between 2009 and 2020. *Lycalopex griseus* exhibited a sharp increase in case numbers between 2014 and 2018, peaking in 2017, a pattern also observed for *Lycalopex culpaeus*, which reached its maximum in the same year. Isolated reports were recorded for *Lycalopex sechurae* in 2022.

## DISCUSSION

Our review indicates that sarcoptic mange is widespread among South American canids, affecting multiple species across a broad geographic range. However, published cases likely underestimate the true occurrence of the disease. For instance, we found no recent reports of mange in canids from Argentina (except Fain, 1968), despite several documented outbreaks in wild camelids across the country (Martin et al., 2023). Additionally, relevant records of scabies in Neotropical canids may be overlooked in systematic reviews when published in non‐indexed or specialized journals, limiting their visibility in commonly used bibliographic databases, as observed in the present study.

The higher number of mange records in dry climates may result from a combination of ecological and observational factors. Sparse vegetation increases the detectability of symptomatic animals, while concentrated water and food resources may promote aggregation, facilitating mite transmission. Furthermore, many dry areas coincide with anthropized landscapes where domestic dogs interact with wildlife, potentially increasing reporting rates.

Although anecdotal, our review suggests a possible association between mange occurrence and anthropized landscapes, particularly areas dominated by agriculture and cattle ranching. This pattern may indicate that land‐use changes may elevate contact between domestic dogs and wildlife, promoting cross‐species transmission (Fiorello et al., 2006; Fiori et al., 2025). A similar pattern has been reported for culpaeos (*Lycalopex culpaeus*), in which most infected individuals were recorded near human dwellings (Millán et al., 2024). Similar trends have been reported in North American coyotes (*Canis latrans*), where individuals with mange were more likely to frequent residential areas and compost piles (Murray et al., 2015; Murray & St. Clair, 2017). Although some cases occurred in relatively isolated regions, previous research indicates that wild canids remain susceptible to infectious and parasitic agents from domestic dogs even in remote habitats (Deem & Emmons, 2005; Ellwanger & Chies, 2019; Fiorello et al., 2006).

In total, 28 animals were treated and recovered from mange, yet the overall effectiveness of such interventions remains uncertain. Most reports of successful sarcoptic mange treatment in wild canids concern isolated cases rather than population‐level control efforts (Jorge et al., 2008; Moroni et al., 2020; Rowe et al., 2019; Van Wick et al., 2020). As observed in our study of Neotropical canids, ivermectin is the most commonly used treatment in other mammal species as well (Rowe et al., 2019), but orally ingested fluralaner showed promising use for raccoon dogs (*Nyctereutes procyonoides*), a Eurasian canid (Hyun et al., 2019). As emphasized by Niedringhaus et al. (2019), strategies for managing mange in free‐ranging wildlife without direct treatment are still poorly explored, and killing of diseased individuals do not seem to reduce prevalence. Similarly, Fiori et al. (2025) noted that the efficacy of population‐level control or eradication remains inconclusive, as research has largely focused on individual recovery rather than the broader ecological and environmental implications of antiparasitic use. This concern is not restricted to Neotropical wild canids but has also been highlighted in other species, such as Arctic foxes (*Vulpes lagopus*) and raccoon dogs (*Nyctereutes procyonoides*) (Hyun et al., 2019; Wallén et al., 2024). Nevertheless, short‐term treatment in *V. lagopus* has shown promising results, as foxes associated with several treated dens showed signs of recovery and subsequently produced litters (Wallén et al., 2024). Preventive actions should therefore be prioritized, including reducing artificial feeding, enforcing strict biosecurity during wildlife handling and transport, and, when possible, treating and rehabilitating affected individuals. Integrating disease surveillance into wildlife management plans may improve early detection, particularly when combined with coordinated efforts among veterinary agencies, protected‐area management and local communities.

A common limitation across the analysed studies is that skin scrapings were generally not performed on apparently healthy individuals, which likely leads to underestimation of disease prevalence. We therefore recommend that future studies include examinations of clinically healthy animals to obtain a more accurate picture of infection rates. Our review also reveals a sharp increase in recorded cases after 2014 (Figure 1d). However, this pattern should be interpreted with caution, as it may reflect an increase in research and publication effort rather than a true rise in the number of cases or in disease prevalence.

Beyond individual morbidity, sarcoptic mange may influence population dynamics by reducing reproductive success or altering predator–prey relationships. Severe population declines, and even local extirpations, have been observed in red fox (*Vulpes vulpes*) populations in Europe, which in turn affected the abundance of prey species such as roe deer (*Capreolus capreolus*) and black grouse (*Lyrurus tetrix*) (Lindström et al., 1994; Mörner, 1992). If similar dynamics occur in the Neotropics, mange‐induced declines in populations of one of its largest predators, the maned wolf (*Chrysocyon brachyurus*), could affect populations of several small mammals that constitute its prey (Motta‐Junior et al., 1996; Rodrigues et al., 2007). Likewise, in small or fragmented populations, such as those of *Speothos venaticus*, recurrent outbreaks could accelerate local declines and erode genetic diversity, highlighting the potential conservation implications of the disease.

Interspecific transmission, however, remains poorly understood, and existing studies have not reached a consensus. Verdugo et al. (2016) reported that mites from a *Lycalopex griseus* specimen in Chile were genetically similar to those from European rabbits (*Oryctolagus cuniculus*). Similarly, Gomez‐Puerta et al. (2024) detected closely related DNA sequences between mites from *Lycalopex culpaeus* and, respectively, domestic dogs and wild camelids in Peru. Astorga et al. (2025) also found some association between domestic dogs and species of *Lycalopex* affected by mange. In Cerrado areas of southeastern Brazil, Fiori et al. (2023) suggested that domestic dogs, as well as exotic boars (*Sus scrofa*) and hares (*Lepus europaeus*), could act as potential sources of transmission, highlighting the complexity of host–parasite interactions in human‐modified landscapes. It is therefore plausible that these transmission pathways are not mutually exclusive and that a wide variety of mammalian hosts, both wild and domestic, may contribute to the maintenance and spread of *Sarcoptes scabiei*.

In summary, sarcoptic mange poses a growing threat to native South American canids, reflecting broader challenges at the wildlife‐livestock interface. Expanding interdisciplinary research that links ecology, genomics and management is crucial to prevent further spillover and safeguard vulnerable carnivore populations across the Neotropics.

## AUTHOR CONTRIBUTIONS

**Luan de Jesus Matos de Brito:** Conceptualization; investigation; writing – original draft; methodology; validation; writing – review and editing; funding acquisition; formal analysis; data curation. **Guilherme S. T. Garbino:** Writing – review and editing; investigation; project administration; writing – original draft; visualization; data curation.

## CONFLICT OF INTEREST STATEMENT

The authors declare no conflicts of interest.

## Supporting information


**Figure S1.** Flowchart summarizing the literature screening process for the systematic review on scabies in Neotropical canids.

## Data Availability

The data that support the findings of this study are openly available in Zenodo at https://doi.org/10.5281/zenodo.18962597.
