# Supplementary material for: Occurrence and distribution of sarcoptic mange in wild Neotropical canids
Source: Med Vet Entomol. 2026 May 4;40(4):1106–14. doi: 10.1111/mve.70085 (PMC13749596; doi:10.1111/mve.70085)
Supplement: Supplementary file 1 [file MVE-40-1106-s001.pdf]

SUPPLEMENTARY MATERIAL

Occurrence and distribution of sarcoptic mange in wild Neotropical canids

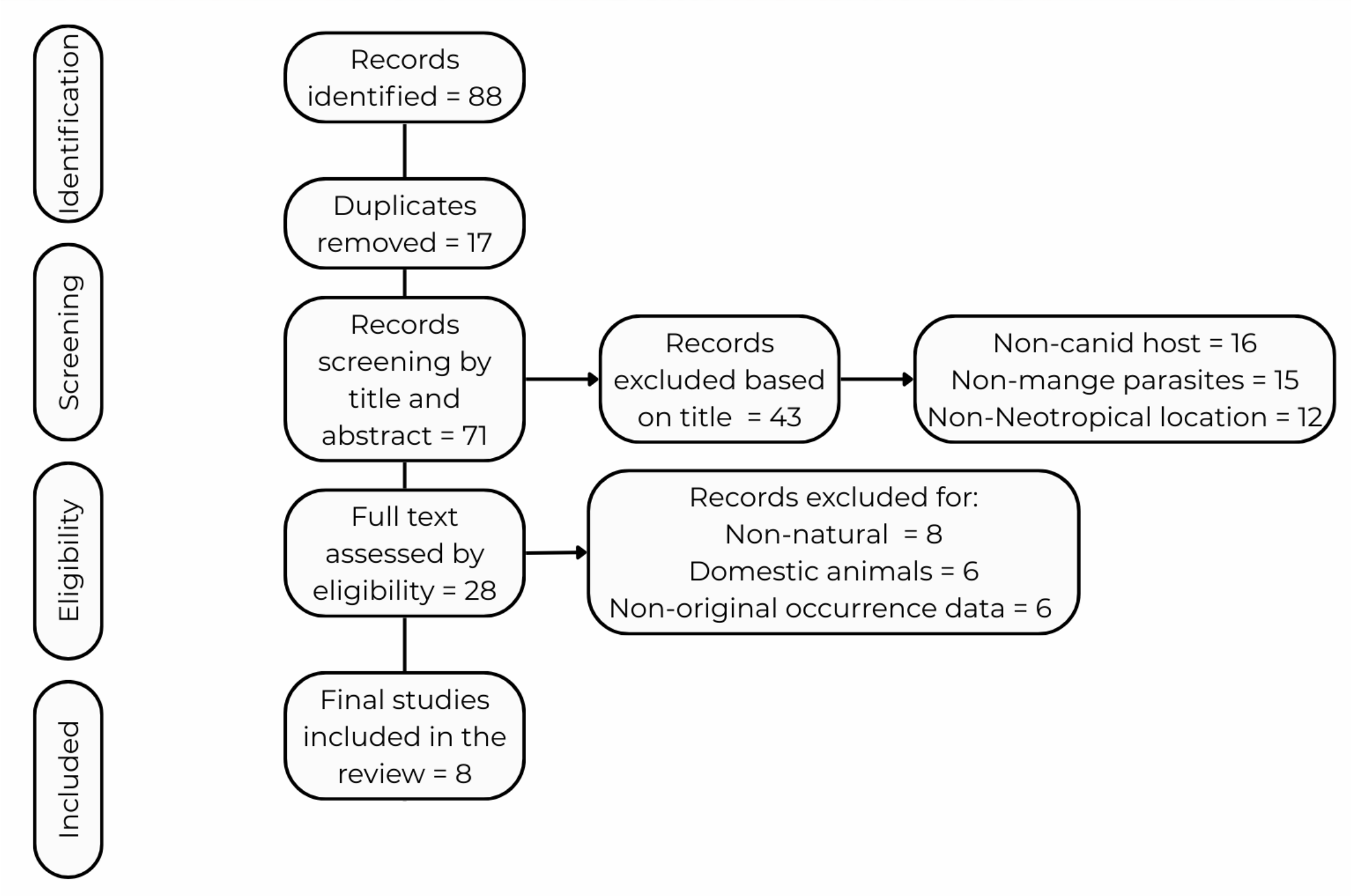

Figure S1. Flowchart summarizing the literature screening process for the systematic review on scabies in Neotropical canids.
